# Redo‐TAVR for bioprosthetic valve degeneration with obvious neoplasm in a left cerebral infarction patient

**DOI:** 10.1002/ccr3.9315

**Published:** 2024-08-07

**Authors:** Weili Liu, Dacheng Li, Zhanjun Qu, Guozhang Tang, Song Liu, Yanchao Li, Lei Jiang

**Affiliations:** ^1^ Interventional Operation Room Affiliated Hospital of Qingdao University Qingdao China; ^2^ Department of Nuclear Medicine Affiliated Hospital of Qingdao University Qingdao China; ^3^ Department of Cardiovascular Surgery Affiliated Hospital of Qingdao University Qingdao China; ^4^ Department of Echocardiography Affiliated Hospital of Qingdao University Qingdao China; ^5^ Department of Cardiovascularology Affiliated Hospital of Qingdao University Qingdao China

**Keywords:** bioprosthetic valve degeneration, cerebral infarction, neoplasm, redo‐transcatheter aortic valve replacement (redo‐TAVR)

## Abstract

**Key Clinical Message:**

In recent years, it is necessary to Redo‐TAVR for the patients with bioprosthetic valve degeneration. This case report described a unique instance to successfully Redo‐TAVR a patient with bioprosthetic valve degeneration, in addition, with left cerebral infarction and renal insufficiency.

**Abstract:**

Over time, more and more patients have bioprosthetic valve degeneration either used in SAVR or TAVR. In order to solve the produced problems due to the degenerated bioprosthetic valve, Redo‐TAVR was increasingly popular due to its safe and efficiency especially for the high risk and complicated symptoms patients. In this case, the patient with left cerebral infarction and renal insufficiency has exhibited severe regurgitation and obvious neoplasm around the previous replaced aortic valve. For the patient with complicated symptoms, we did not image for this patient and only used CT to determine the position and angle for the Redo‐TAVR on the base of metal stent for the previous replaced aortic valve. During the Redo‐TAVR process, for fear of the obvious neoplasm slipping from the previous replaced aortic valve to embolism of important organs, before carrying out the Redo‐TAVR, cerebral protection device, temporary pacemaker, and coronary artery protection device were utilized in order to avoid the damage for the important organs from the obvious neoplasm slipping from the previous replaced aortic valve. The surgery was successful and the patient recovered well. The patient's symptoms of chest tightness and suffocation have been greatly reduced.

## 1 INTRODUCTION

In recent years, the proportion of bioprosthetic valve utilization for heart diseases has been continuously increasing. However, bioprosthetic valve replacement is associated with degeneration which might be due to passage of time and various factors such as leaflet degeneration or non‐leaflet degeneration.^1^, ^2^ The leaflet degeneration is generally caused by valve leaflet wear, tear or calcification, and valve leaflet damage (endocarditis), which is mainly caused by blood vessels pancreatic coverage, thrombosis, and perivalvular leakage.^3^ Therefore, it is necessary to redo biosprosthetic valve replacement. Presently, there are two main methods to redo aortic valve including redo‐surgical aortic valve replacement (Redo‐SAVR) and Redo‐Transcatheter aortic valve replacement (Redo‐TAVR). Redo‐SAVR has been the main treatment method of degenerated aortic bioprothesis. However, due to various factors such as advanced age, comorbidities and redo‐sternotomy etc. for patients, it is related with higher surgical risk than the first SAVR.^4^, ^5^ Comparatively, Redo‐TAVR as a better treatment technique has gained more and more popularity especially in high risk patients for Redo‐SAVR, because it decreases the morbidity and mortality, and produces favorable outcomes.^6^ Therefore, it is a suitable option for degenerated bioprosthetic valves. Here, we present a Redo‐TAVR case of 69‐year‐old man with obvious neoplasm in the degenerated bioprosthetic valve for a left cerebral infarction patient.

## 2 CASE HISTORY

A 69‐year‐old patient with a previous surgical aortic valve (A‐25, BalMedic) replacement 11 years ago because of aortic insufficiency was admitted to the hospital due to obvious symptom with chest tightness and short of breath for about 2 months and aggravated 1 week before admission, and accompanied by bilateral lower limb edema. In addition, this patient has 9 years of history of left cerebral infarction and poor mobility on the right side, which made the condition more complex.

## 3 METHODS

**FIGURE 1:**
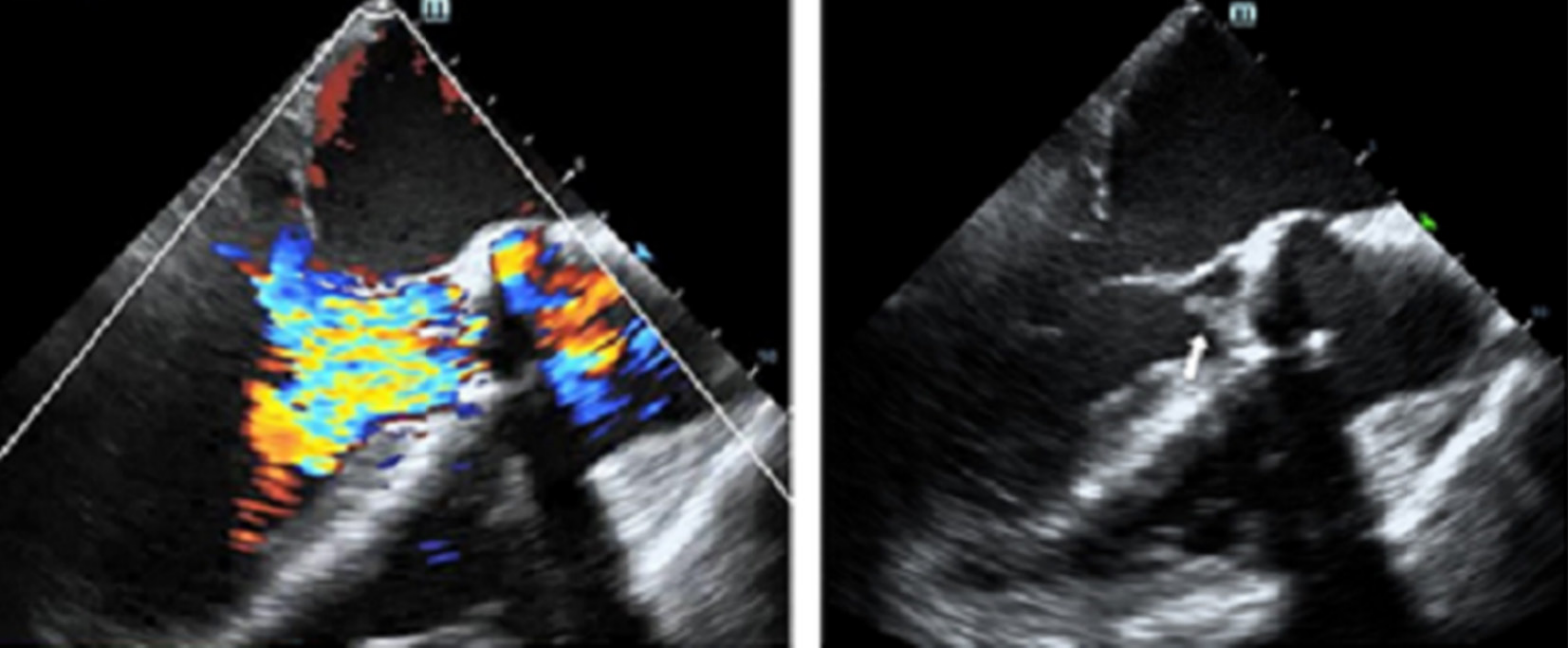
Transesophageal echocardiography before Redo‐TAVR.

The risk score was 14.14% according to EuroSCORE II which showed high risk for the patient. Then, transesophageal echocardiography was carried out to determine the patient. The result showed that the previous replaced aortic valve produced severe regurgitation, moderate pulmonary hypertension, and obvious neoplasm has formed around the aortic valve (See Figure 1). Additionally, blood cultures were negative not only under anaerobic conditions but also under aerobic conditions, which indicated that the patient has no infective endocarditis. Considering the above complex conditions and wishes of the patient, our multidisciplinary structural heart team thought that Redo‐SAVR method was high risk and Redo‐TAVR was suitable for this patient.

**FIGURE 2:**
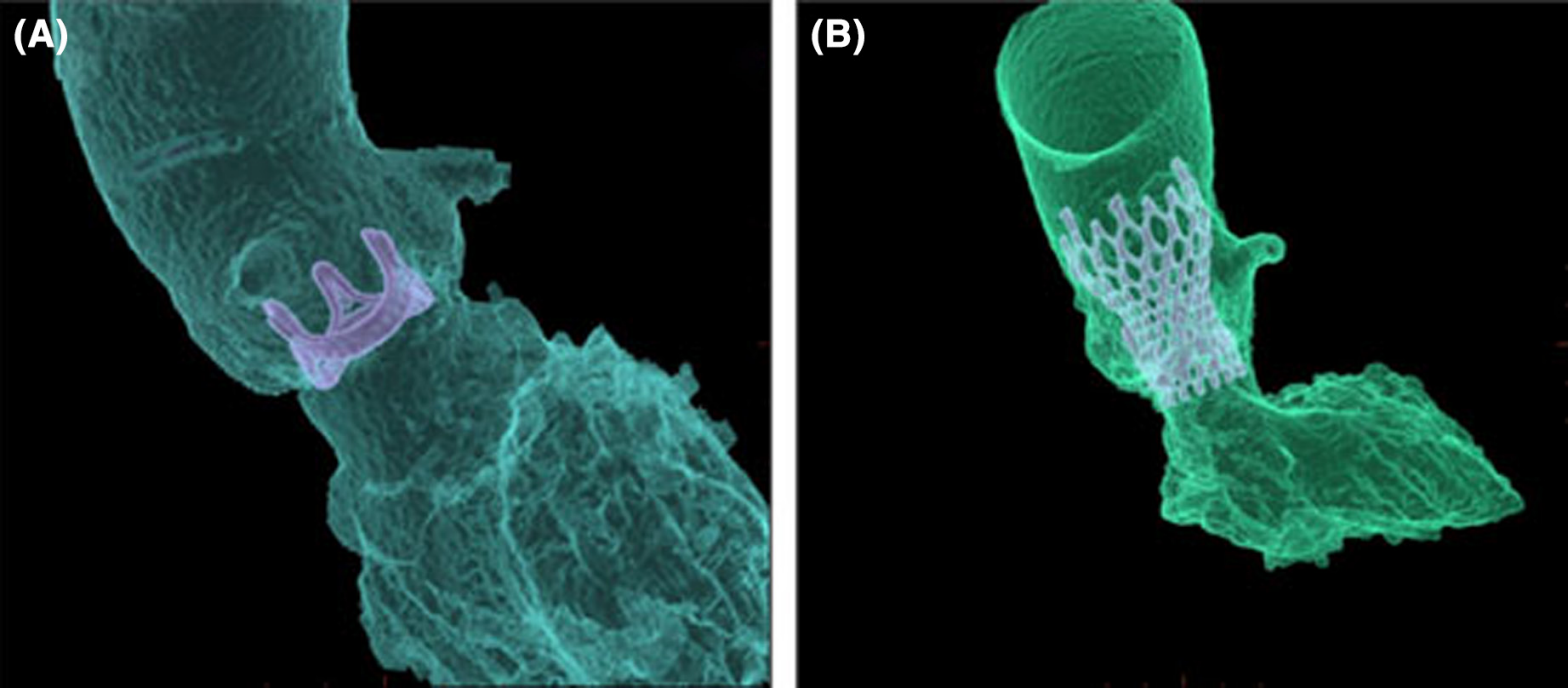
CT three‐dimensional reconstruction of aortic root (A) Preoperation; (B) postoperation.

**FIGURE 3:**
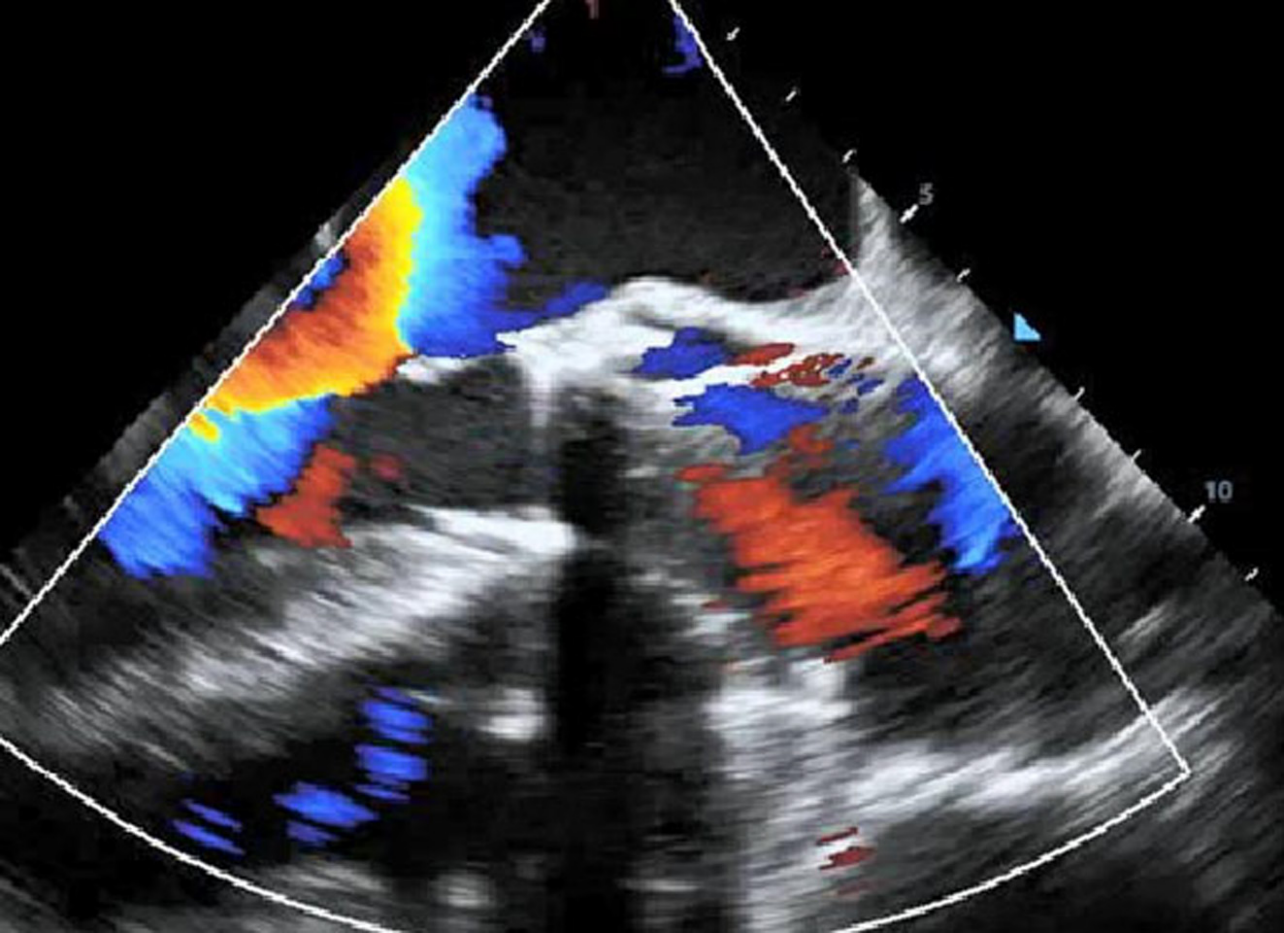
Transesophageal echocardiography After Redo‐TAVR.

Firstly, because the previous replaced aortic valve had metal stent, we used computed tomography angiography (CTA) to determine position and angle of Redo‐TAVR without usage of contrast agent considering obvious neoplasm around the aortic valve(see Figure 2A). Similarly, for fear of the obvious neoplasm slipping from the previous replaced aortic valve to embolism of important organs, before carrying out the Redo‐TAVR, cerebral protection device, temporary pacemaker, and coronary artery protection device were utilized. Briefly, temporary pacemaker was implanted through the right jugular vein and it paced effectively. Because the patient had a previous left cerebral infarction, the right cerebral artery needed protection. An ev3 SpiderFX 5.0 mm protection device was inserted into the right internal carotid artery. In addition, in order to perform coronary artery protection, loach guide wire was used to guide 6F JL 3.5 to the left coronary artery opening, and HI‐TORQUE guided to extend the catheter to the proximal end of the left anterior descending branch. According to the results of CTA, Venus‐A Plus 26(Venus Medtech (Hangzhou) Inc.) was implanted at the predetermined accurate location and rapidly paced. The position and morphology of the valve were satisfactory under fluoroscopy (see Figure 2B). Furtherly, through transesophageal echocardiography, it was found that the artificial valve had a good shape, and there was no obvious intra valve regurgitation or perivalvular leak (see Figure 3). Then, the coronary protection device and cerebral artery protection device were withdrawn. The result of echocardiography showed that the valve function was normal. Additionally, and the patient's vital signs were stable after Redo‐SAVR. The distal pulsation of both lower limb arteries was good. During hospitalization, anticoagulation therapy was initiated with rivaroxaban. After 7 days in hospital, the patient was discharged home and was instructed to continue rivaroxaban to reduce the stroke risk. Furthermore, we followed up the patient for 3 months to observe his symptoms. And the results of electrocardiogram after 1 and 3 months showed that the implanted valve of the patient worked well.

## 4 CONCLUSION

For patients with degenerated bioprosthetic valves especially for the high risk and complicated symptoms patients, it is crucial to choose a safe and efficient treatment method to replace the bioprosthetic valves again. Redo‐TAVR as a better treatment technique has gained more and more popularity because of the reduced the morbidity and mortality, and producing favorable outcomes. Therefore, it is a suitable option for degenerated bioprosthetic valves. In this case, the patient has exhibited severe regurgitation and obvious neoplasm around the previous bioprosthetic aortic valve. Furthermore, he suffered the left cerebral infarction for 9 years and had renal insufficiency. Therefore, he was a complicated symptom with high risk. For the special patient, we successfully carried out Redo‐TAVR without preoperative image, with intraoperative cerebral protection and coronary artery protection. The patient recovered well. After 1 and 3 months the results of echocardiography also showed that the implanted valve of the patient worked well. This case provides reference for other similar cases.

## 5 DISCUSSION

With passage of time, more and more patients with bioprosthetic valve degeneration either used in SAVR or TAVR occurred. At present, the lifespan of bioprosthetic valves is about 15 years, and 30% to 60% of the valves may degenerate after expiration. In clinical practice, secondary valve replacement is required after the failure of bioprosthetic valves, and surgical thoracotomy often faces greater difficulty and risk than the first surgery. The mortality rate of high‐risk patients undergoing surgical valve replacement again exceeds 20%.^7^ Since the first TAVR was performed by Cribier et al in 2002, it has been proven its efficacy and benefit in terms of mortality and improvement in quality of life by numerous studies.^8^, ^9^ Thus, in order to solve the produced problems due to the degenerated bioprosthetic valve, Redo‐TAVR was increasingly popular due to its minimally invasive procedure especially for the high risk and complicated symptoms patients. In this case, the patient has exhibited severe regurgitation and obvious neoplasm around the aortic valve. In addition, he has renal insufficiency (Urea 14.60 mmol/L; Creatinine 126umol/L). Before Redo‐TAVR, it is common to image the position and size of the valve. However, for this case, the imaging process was accompanied with high pressure which was easy to cause the neoplasm slip from the original valve so it might damage important organs. In addition, the contrast agent might aggravate his renal insufficiency. Therefore, we did not image for this patient and only used CT to determine the position and angle for the Redo‐TAVR on the base of metal stent for the previous replaced aortic valve.

On the other hand, one of the most devastating TAVR‐related complications is stroke, which will severely impair the life quality and mortality of the patients, although the reported rate was low.^10^, ^11^, ^12^ To avoid stroke and protect the supra‐aortal arteries from embolized material during the procedure of TAVR, mechanical cerebral protection systems have been developed. Carolina et al. showed that a Sentinel™ Cerebral Protection System (CPS; Boston Scientific, Marlborough, MA, USA) could lower rates of cerebrovascular events, shortened length of hospital stay, and improved 12‐month survival of the patients of TAVR.^13^ Similar results were exhibited by a larger and retrospective analysis of 1305 TAVR patients. The application of Sentinel™ was related with a reduction of cerebrovascular events at 72 h of 65% (*p* < 0.01) and a reduction of the combined endpoint consisting of all‐cause mortality and cerebrovascular events at 72 h of 66% (*p* < 0.01).^14^ In this study, the patient suffered from left cerebral infarction and still had poor mobility on the right side. If cerebrovascular embolism occurred due to the neoplasm slipping from the original valve, it was more likely to have another cerebral infarction which would severely affected the quality of life and even the survival rate of the patient. Therefore, it was necessary to carry out cerebral protection technique during the process of Redo‐TAVR.

Besides cerebrovascular embolism, Redo‐TAVR often results in the coronary obstruction, which is a major concern regarding the feasibility of the technique.^15^ In this situation, some protective coronary techniques had been used for feasibility of Redo‐TAVR. For example, the BASILICA, a leaflet laceration technique, is an efficient method to avoid coronary obstruction. It was successfully used in several Redo‐TAVR cases.^16^ In addition, preventive stenting and wires were also successfully used in some cases. Palmerini et al. reported the safety and efficacy of coronary protection by pre‐emptive wiring and provisional stenting of coronary arteries in 236 patients for 3‐year clinical outcomes at high risk for coronary obstruction undergoing TAVR.^17^ In this case, due to the occurrence of the neoplasm around the original valve, in order to avoid the coronary obstruction, we used a wiring to protect coronary artery for the process of Redo‐TAVR. And after the surgery, the wiring was withdrawn without effect to the patient.

## AUTHOR CONTRIBUTIONS

**Weili Liu:** Conceptualization; writing – original draft. **Dacheng Li:** Methodology; writing – original draft. **Zhanjun Qu:** Methodology; writing – original draft. **Guozhang Tang:** Methodology. **Song Liu:** Methodology. **Yanchao Li:** Methodology; writing – original draft. **Lei Jiang:** Project administration; writing – review and editing.

## FUNDING INFORMATION

The author(s) received no financial support for the research, authorship, and/or publication of this article.

## CONFLICT OF INTEREST STATEMENT

The author(s) declared no potential conflicts of interest with respect to the research, authorship, and/or publication of this article.

## ETHICS STATEMENT

Ethical approval was not required for this study in accordance with local or national guidelines.

## CONSENT

Written informed consent was obtained from the patient to publish this report in accordance with the journal's patient consent policy.

## Data Availability

The data that support the findings of this study are available from the corresponding author upon reasonable request.

## References

[1] Saleem S , Ullah W , Syed MA , et al. Meta‐analysis comparing valve‐in‐valve TAVR and redo‐SAVR in patients with degenerated bioprosthetic aortic valve. Catheter Cardiovasc Interv. 2021;98:940‐947. doi:10.1002/ccd.29789 34110684

[2] Grube E , Sinning JM . The “big five” complications after transcatheter aortic valve replacement: do we still have to be afraid of them? JACC Cardiovasc Interv. 2019;12(4):370‐372. doi:10.1016/J.JCIN.2018.12.019 30784642

[3] Rahimtoola SH . Choice of prosthetic heart valve in adults: an update. J Am Coll Cardiol. 2010;55(22):2413‐2426. doi:10.1016/j.jacc.2009.10.085 20510209

[4] Baumgartner H , Falk V , Bax JJ , de Bonis M , Hamm C . ESC/EACTS guidelines for the management of valvular heart disease. Eur Heart J. 2017;38(36):2739‐2791. doi:10.1093/eurheartj/ehac051 28886619

[5] Maganti M , Rao V , Armstrong S , Feindel CM , Scully HE , David TE . Redo valvular surgery in elderly patients. Ann Thorac Surg. 2009;87(2):521‐525. doi:10.1016/j.athoracsur.2008.09.030 19161771

[6] Carabello BA . Transcatheter aortic‐valve implantation for aortic stenosis in patients who cannot undergo surgery. Curr Cardiol Rep. 2011;13(3):173‐174. doi:10.1056/NEJMOA1008232 21336623

[7] Schoen FJ , Levy RJ . Calcification of tissue heart valve substitutes: progress toward understanding and prevention. Ann Thorac Surg. 2005;79:1072‐1080. doi:10.1016/j.athoracsur.2004.06.033 15734452

[8] Cribier A , Eltchaninoff H , Bash A , et al. Percutaneous transcatheter implantation of an aortic valve prosthesis for calcific aortic stenosis: first human case description. Circulation. 2002;106:3006‐3008. doi:10.1016/S1062-1458(03)00094-1 12473543

[9] Devireddy C , Hiremath S . Acute kidney injury after transcatheter aortic valve replacement. Circ Cardiovasc Interv. 2018;11(8):e007135. doi:10.1111/jocs.12768 30354790

[10] Tay ELW , Gurvitch R , Wijesinghe N , et al. A high‐risk period for cerebrovascular events exists after transcatheter aortic valve implantation. JACC Cardiovasc Interv. 2011;4:1290‐1297. doi:10.1016/j.jcin.2011.08.012 22192370

[11] Mack MJ , Leon MB , Thourani VH , et al. Transcatheter aortic‐valve replacement with a balloon‐expandable valve in low‐risk patients. N Engl J Med. 2019;380:1695‐1705. doi:10.1056/NEJMoa1814052 30883058

[12] Smith CR , Leon MB , Mack MJ , et al. Transcatheter versus surgical aortic‐valve replacement in high‐risk patients. N Engl J Med. 2011;364:2187‐2198. doi:10.1056/NEJMoa1103510 21639811

[13] Carolina D , Matthias K , Christian N , et al. Cerebral protection in TAVR—can we do without? A real‐world all‐comer intention‐to‐treat study—impact on stroke rate, length of hospital stay, and twelve‐month mortality. J Pers Med. 2022;12:320. doi:10.3390/jpm12020320 35207808 PMC8878932

[14] Seeger J , Kapadia SR , Kodali S , et al. Rate of peri‐procedural stroke observed with cerebral embolic protection during transcatheter aortic valve replacement: a patient‐level propensity‐matched analysis. Eur Heart J. 2019;40:1334‐1340. doi:10.1093/eurheartj/ehy847 30590554

[15] Chen F , Xiong T , Li Y , et al. Risk of coronary obstruction during redo‐TAVR in patients with bicuspid versus tricuspid aortic valve stenosis. JACC Cardiovasc Interv. 2022;15(7):712‐724. doi:10.1016/j.jcin.2022.01.282 35393104

[16] Lederman RJ , Babaliaros VC , Rogers T , et al. Preventing coronary obstruction during transcatheter aortic valve replacement: from computed tomography to BASILICA. J Am Coll Cardiol Intv. 2019;12:1197‐1216. doi:10.1016/j.jcin.2019.04.052 PMC6724191 31272666

[17] Palmerini T , Chakravarty T , Saia F , et al. Coronary protection to prevent coronary obstruction during TAVR: a multicenter international registry. J Am Coll Cardiol Intv. 2020;13:739‐747. doi:10.1016/j.jcin.2019.11.024 32061608

